# Efficacy of a Plant-Microbe System: *Pisum sativum* (L.) Cadmium-Tolerant Mutant and *Rhizobium leguminosarum* Strains, Expressing Pea Metallothionein Genes *PsMT1* and *PsMT2*, for Cadmium Phytoremediation

**DOI:** 10.3389/fmicb.2020.00015

**Published:** 2020-01-29

**Authors:** Viktor E. Tsyganov, Anna V. Tsyganova, Artemii P. Gorshkov, Elena V. Seliverstova, Viktoria E. Kim, Elena P. Chizhevskaya, Andrey A. Belimov, Tatiana A. Serova, Kira A. Ivanova, Olga A. Kulaeva, Pyotr G. Kusakin, Anna B. Kitaeva, Igor A. Tikhonovich

**Affiliations:** ^1^All-Russian Research Institute for Agricultural Microbiology, Saint Petersburg, Russia; ^2^Saint Petersburg Scientific Center (RAS), Saint Petersburg, Russia; ^3^Sechenov Institute of Evolutionary Physiology and Biochemistry (RAS), Saint Petersburg, Russia; ^4^Department of Genetics and Biotechnology, Saint Petersburg State University, Saint Petersburg, Russia

**Keywords:** symbiotic engineering, cadmium tolerance, nodulation, metallothionein, bacteroid, symbiosome, *Rhizobium*

## Abstract

Two transgenic strains of *Rhizobium leguminosarum* bv. *viciae*, 3841-PsMT1 and 3841-PsMT2, were obtained. These strains contain the genetic constructions *nifH-PsMT1* and *nifH-PsMT2* coding for two pea (*Pisum sativum* L.) metallothionein genes, *PsMT1* and *PsMT2*, fused with the promoter region of the *nifH* gene. The ability of both transgenic strains to form nodules on roots of the pea wild-type SGE and the mutant SGECd^t^, which is characterized by increased tolerance to and accumulation of cadmium (Cd) in plants, was analyzed. Without Cd treatment, the wild type and mutant SGECd^t^ inoculated with *R. leguminosarum* strains 3841, 3841-PsMT1, or 3841-PsMT2 were similar histologically and in their ultrastructural organization of nodules. Nodules of wild-type SGE inoculated with strain 3841 and exposed to 0.5 μM CdCl_2_ were characterized by an enlarged senescence zone. It was in stark contrast to Cd-treated nodules of the mutant SGECd^t^ that maintained their proper organization. Cadmium treatment of either wild-type SGE or mutant SGECd^t^ did not cause significant alterations in histological organization of nodules formed by strains 3841-PsMT1 and 3841-PsMT2. Although some abnormalities were observed at the ultrastructural level, they were less pronounced in the nodules of strain 3841-PsMT1 than in those formed by 3841-PsMT2. Both transgenic strains also differed in their effects on pea plant growth and the Cd and nutrient contents in shoots. In our opinion, combination of Cd-tolerant mutant SGECd^t^ and the strains 3841-PsMT1 or 3841-PsMT2 may be used as an original model for study of Cd tolerance mechanisms in legume-rhizobial symbiosis and possibilities for its application in phytoremediation or phytostabilization technologies.

## Introduction

Plant-microbial systems formed by legumes and soil bacteria, called rhizobia, are widely used to enrich soils with nitrogen (Stagnari et al., 2017). Nitrogen fixation takes place in symbiotic nodules formed on plant roots (and in some cases on shoots), and this nodule formation creates a new ecological niche for rhizobia. For many legume plants, rhizobia use the infection thread to penetrate inside their roots (Tsyganova and Tsyganov, 2018). From these infection threads, rhizobia are released into host cells’ cytoplasm where they differentiate into bacteroids and form symbiosomes, the main units fixing nitrogen. Simultaneously, infected plant cells may become differentiated during this process, thus enabling them to host numerous symbiosomes (Tsyganova et al., 2018).

The process of nitrogen fixation is sensitive to environmental cues, including the action of heavy metals (Angle et al., 1993; Neumann et al., 1998). Cadmium (Cd) is one of the most toxic elements and plants use different mechanisms to mitigate its toxic effects (Kulaeva and Tsyganov, 2011). The presence of Cd in the soil often reduces the formation of nodules and inhibits their functioning. For example, in *Lupinus albus* L., the biomass of nodules from plants treated with Cd reached just 57% of that attained by control plants, having an average nodule weight that decreased by 51% while their total nitrogen content decreased by 32% (Carpena et al., 2003). In *Medicago sativa* L. plants treated with Cd, nodule development was delayed (Ibekwe et al., 1996). Negative effects of Cd on nitrogen fixation were also demonstrated for nodules of *Pisum sativum* L. (Hernandez et al., 1995), *Glycine max* (L.) Merr. (Balestrasse et al., 2004), *L. albus* (Sánchez-Pardo et al., 2013), *M. sativa* L. (Shvaleva et al., 2010), *M. truncatula* Gaertn. (Marino et al., 2013), and in some other species too. Cd also affects nodule ultrastructure, leading to accumulated glycoproteins in the intercellular space, accumulation of insoluble Cd in the cell wall, and degradation of bacteroids (Carpena et al., 2003). In *P. sativum* nodules exposed to Cd, expansion of the peribacteroid space, destruction of the symbiosome membrane, fusion of symbiosomes, and the formation of symbiosomes containing several bacteroids were all observed (Tsyganova et al., 2019). Similar features of Cd’s adverse influence on nodule ultrastructure were reported for nodules of *M. sativa* (Shvaleva et al., 2010).

Despite the high sensitivity of nodule development and functioning to Cd, legume-rhizobial systems have only recently been considered as a promising tool for soil phytoremediation, including Cd-contaminated soils (Safronova et al., 2011; Hao et al., 2014; Pajuelo et al., 2014; Gómez-Sagasti and Marino, 2015; Teng et al., 2015; Fagorzi et al., 2018). The advantages of rhizobia include their rapid growth and being isolated in a nodule, where Cd is accumulated and then transported to aboveground parts of the host plant. Legumes are also characterized by fast growth and the accumulation of large biomass. Yet, to take full advantage of legume-rhizobial systems for phytoremediation applications, it is crucial to select plant and bacterial genotypes with increased tolerance to Cd. In general, rhizobia are much more tolerant to Cd than are legumes (Belimov et al., 2015b). Thus, selecting appropriate plant genotypes tolerant to Cd is paramount for developing plant-microbial systems for Cd phytoremediation.

In pea, the mutant SGECd^t^ (*cdt*) displayed an increased tolerance of Cd and accumulated it in excess amounts (Tsyganov et al., 2007). The mutation was localized on its genetic map, but the gene has yet to be identified (Kulaeva and Tsyganov, 2013; Tsyganov et al., 2013). The mutation *cdt* increases the tolerance of nodulation of mutant plants to Cd in comparison with wild-type plants (Tsyganov et al., 2005). Recently, it was shown that nodules in the mutant SGECd^t^ demonstrate an increased tolerance of short exposure to high Cd concentrations (100 μM and 1 mM of CdCl_2_) when compared with wild-type SGE nodules (Tsyganova et al., 2019).

Despite rhizobia having higher tolerance than legumes to Cd, their genetic modification allows the introduction of additional tolerance not observed in the original strain. The use of legumes and transgenic strains of rhizobia for phytoremediation, called “symbiotic engineering,” was suggested just over 10 years ago (Sriprang and Murooka, 2007). Its first successful experiment was the construction of a transgenic—carrying the gene of human metallothionein *MTL4*—strain of *Mesorhizobium huakuii* subsp. *rengei*, which formed nodules with *Astragalus sinicus* L. (Sriprang et al., 2002). The *MTL4* gene was fused with the promoters of *nifH* and *nolB* genes of *M. huakuii*, which ensured its expression both in nodule bacteria cells and in symbiotic nodules. Using the obtained strain in symbiosis with *A. sinicus* led to a 6-fold increase in Cd accumulation in its nodules (Sriprang et al., 2002). Importantly, it was estimated that one nodule could accumulate 1.4 nM of Cd, which for 100 nodules per plant on average, this amounts to 140 nM captured per plant (Sriprang and Murooka, 2007). A transgenic strain carrying the *Arabidopsis thaliana PCS* gene, encoding phytochelatin synthase, has also been obtained, for which a 9–19-fold increase in the Cd content of free-living rhizobia was observed, accompanied by a 1.5-fold increase in symbiotic nodules (Sriprang et al., 2003). However, the construction of transgenic strains carrying both *MTL4* and *PCS* genes could enable further increases in the Cd content of nodules when compared with recombinant strains carrying only one of these genes. Moreover, such a strain has already been successfully applied for phytoremediation of rice fields, in that 9% of Cd was removed from the soil within 2 months of cultivation (Ike et al., 2007). In addition to transgenic strains resistant to Cd, a genetically modified *Ensifer medicae* strain MA11 expressing copper tolerance genes *copAB* was obtained (Delgadillo et al., 2015). More recently, a double genetically modified symbiotic system was created for the phytostabilization of copper in the roots of the legume plant *M. truncatula*. This system included composite plants of *M. truncatula*, in which the metallothionein gene *MT4a* from *A. thaliana* was expressed in transformed hairy roots and a transgenic strain *E. medicae* that expressed the *copAB* genes (Pérez-Palacios et al., 2017). This double symbiotic system enabled an increase in tolerance to copper and fostered elevated levels of copper in roots. However, the translocation of copper from roots to shoots was decreased in plants inoculated with the transgenic strain MA11-*copAB*; hence, this double symbiotic system was deemed appropriate for copper phytostabilization (Pérez-Palacios et al., 2017).

Among the most used genes in “symbiotic engineering” are those of metallothioneins, small cysteine-rich proteins involved in metal detoxification processes and homeostasis (Joshi et al., 2016). Plant metallothioneins are also involved in responses to other different stresses including salt and oxidative stress, pathogen attacks, temperature, and others (Guo et al., 2003). According to the arrangement of cysteine residues, metallothioneins may be subdivided into four types (Cobbett and Goldsbrough, 2002), whose expression is known to display some organ specificity (Joshi et al., 2016). Activation of their expression by Cd has already been demonstrated for various plant species (Guo et al., 2003; Lee et al., 2004; Zhang et al., 2005; Zimeri et al., 2005; Pagani et al., 2012). Thus, metallothionenins clearly play a key role in the detoxification of Cd in plants, and this explains the growing interest in using them for “symbiotic engineering”.

Thus, the aim of this work was to obtain two strains of *R. leguminosarum* bv. *viciae* expressing the pea metallothionein genes *PsMT1* and *PsMT2*, and to investigate formation of symbiotic nodules in the presence of toxic Cd concentrations using Cd-tolerant pea mutant SGECd^t^. Due to the presence of the *nifA* promoter, their expression was activated only in nodules formed on pea plants. According to our knowledge, this is the first example of using genes that are not alien to the symbiotic system for the explicit purpose of “symbiotic engineering.”

## Materials and Methods

### Plant Material

The pea (*Pisum sativum* L.) laboratory line SGE (Kosterin and Rozov, 1993), and its corresponding mutant line SGECd^t^ characterized by an increased tolerance to Cd and higher levels of Cd accumulation (Tsyganov et al., 2007), were both used in this study.

### Bacterial Strain Construction

Two plant genes, encoding metallothioneins of type 1 (AB176564.1) and type 2 (AB176565.1), respectively named *PsMT1* and *PsMT2*, were amplified from *P. sativum* cDNA with the use of these primer pairs: MT1-F/MT1-R (for *PsMT1*) and MT2-F/MT2-R (for *PsMT2*) (Supplementary Table S1). The primers MT1-F and MT2-F contained the first 21 and 22 nucleotide ORFs of *PsMT1* and *PsMT2*, respectively. The sequences for the MT1-R and MT2-R primers were located outside the ORFs of *PsMT1* and *PsMT2* (after the stop codons). Hence, using these primers permitted the PCR-amplification of 268-bp and 264-bp DNA fragments containing the complete ORF of *PsMT1* and *PsMT2*, starting strictly from the start-codons.

The promoter of *nifH* gene was amplified from genomic DNA of *R. leguminosarum* bv. *viciae* by using the primer pair nifH-F/nifH-R. Primer nifH-R consisted of the last 23 nucleotides before the start-codon of the *nifH* gene, while primer nifH-F contained an additionally introduced site for *Bam*HI restrictase. Using primers nifH-F and nifH-R thus enabled amplification of a 750-bp DNA fragment, located strictly in front of the *nifH* gene and containing a promoter.

PCR amplifications were performed using Pfu polymerase (Thermo Fisher Scientific, Waltham, MA, USA) according to standard protocol: initial denaturation at 95°C for 3 min, followed by 30 cycles at denaturation at 94°C for 30 s, primer annealing at 52°C for 30 s, extension at 72°C for 1 min, and a final extension lasting 4 min.

PCR fragments were extracted from agarose gel (Onishchuk et al., 2015), with each *PsMT*-gene ligated with a *nifH* promoter. The obtained ligase mixtures were then used as a matrix in the PCR with primers nifH-F and MT1-R (for fusion with *PsMT1*) or with primers nifH-F and MT2-R (for fusion with *PsMT2*). Each PCR was conducted following the standard technique and using Taq polymerase (Thermo Fisher Scientific). The respectively amplified 1018-bp and 1014-bp fragments were cloned into the pAL-TA vector (Evrogen, Moscow, Russia). The obtained *E. coli* clones were tested *via* PCR with M13-primers. PCR fragments of the corresponding size (approximately 1 kb) were isolated from the gel and sequenced. Sequencing was carried out using the ABI PRISM 3500xl (Applied Biosystems, Waltham, MA, USA) according to the manufacturer’s instructions.

Cloned fragments containing the correct fusions of *PsMT1* and *PsMT2* genes with the *nifH* promoter were restricted by the enzymes *Bam*H1 (included in the nifH-F primer) and *Pst*I (contained in the pAL-TA polylinker) and recloned into the corresponding sites of the vector pCAMBIA 0390 (CAMBIA, Canberra, Australia), which is capable of replication in rhizobia.

In the final step, the obtained *nifH-PsMT1* and *nifH-PsMT2* fusions in the pCAMBIA 0390 plasmid were transferred to the strain 3841 *R. leguminosarum* bv. *viciae* (Wang et al., 1982), by using the method of conjugation (Chizhevskaya et al., 2011). The resulting Km^R^ transconjugants were tested *via* PCR with the primers nifH-F/MT1-R, followed by sequencing of the amplified fragments. The recombinant strains were called 3841-PsMT1 and 3841-PsMT2, respectively, and deposited in the Russian Collection of Agricultural Microorganisms (RCAM 01523 and RCAM 01558, respectively).

### Inoculation and Plant Growth Conditions

Seeds were sterilized with concentrated sulfuric acid for 30 min and rinsed with sterile water 10 times. Seeds were germinated on Petri dishes with moisture filter paper at 24°C in an incubator (Memmert GmbH, Schwabach, Germany). Seven-day-old seedlings were inoculated (1 ml of bacterial suspension containing 10^7^–10^8^ cells per plant) with one of the *R. leguminosarum* bv. *viciae* strains: 3841, 3841-PsMT1, or 3841-PsMT2. Inoculated seedlings were left overnight at 24°C in the incubator. Next, the seedlings were placed on floating rafts in a plastic container (approximately 50 seeds per container) filled with 5 L of a hydroponic solution (μM): NaCl, 5; KH_2_PO_4_, 110; Ca(NO_3_)_2_, 50; MgSO_4_, 400; KCl, 300; CaCl_2_, 70; H_3_BO_3_, 1; MnSO_4_, 1; ZnSO_4_, 1; CuSO_4_, 0.8; Na_2_MoO_4_, 0.03; Fe-tartrate 2.5. The root system was immersed in this solution while the shoots were on the raft’s surface. Each container’s hydroponic solution was supplemented with additional inoculum (bacterial lawn from one Petri dish was used for 5 L of hydroponic solution) and the following day exposed to permanent bubbling. All seedlings were first grown for 3 days, at which point the nutrient solution was replaced and supplemented with 0.5 μM of CdCl_2_, and this hydroponic solution then replaced every 3 days with 0.5 μM of CdCl_2_. All plants were grown in a growth cabinet (a day/night cycle of 16/8 h, at 21°C, 60% relative humidity, and photon flux density during the light phase of 300 mmol quanta m^−2^ s^−1^). Nodules were harvested at 28 day after inoculation.

### Plasmid Stability and Strain Tolerance to Cadmium

Nodules, separated from the roots, were surface sterilized with 96% alcohol for 1 min and homogenized. Serial dilutions were planted on TY agar plates followed by incubation at 28°C for 3 days. The stability of plasmids was estimated by quantifying the number of colonies that maintained antibiotic resistance encoded by the plasmid pCAMBIA. Three independent experiments were performed. The ability of strains to grow under Cd stress was estimated on a liquid mannitol medium containing 5 μM and 10 μM of CdCl_2_.

### Nodule Phenotype Analysis

Photographs of pea nodules were taken with a SteREO Lumar.V12 stereomicroscope equipped with an AxioCam MRc 5 video camera (Zeiss, München, Germany). Object visualization was performed using AxioVision Rel. 4.8 software (Zeiss).

### Expression Analysis

Pea nodules were harvested and ground in liquid nitrogen. Their total RNA extraction and cDNA synthesis were performed as previously described (Serova et al., 2018). The quantity of total RNA was determined using Qubit^™^ RNA Assay Kits on a Qubit® 2.0 Fluorometer (Invitrogen, Waltham, MA, USA).

For gene expression analysis, the primer pairs PsMT-1F*/*PsMT-1R and PsMT-2F/PsMT-2R were used (Supplementary Table S1). Gene-encoding glyceraldehyde-3-phosphate dehydrogenase (*PsGapC1*, L07500.1) served as the reference gene (Ivanova et al., 2015).

Relative real-time PCR was performed using a 10-μl PCR mix (SsoFast Eva Green Supermix (Bio-Rad, Hercules, CA, USA)) in a С1000^™^ Thermal Cycler combined with the optical module CFX96^™^ Real-Time System (Bio-Rad), according to the manufacturer’s protocol. The reaction’s results were processed with Bio-Rad CFX Manager software (Bio-Rad) and analyzed by the 2^−ΔΔCT^ method. Statistical analysis of the experimental results was carried out in GraphPad Prism software.^1^ Statistically significant differences were determined with two-way ANOVA (*p*≤0.05). The experiment was performed in three replicates. For each variant, nodules were collected from several (5–7) plants.

### Light and Electron Microscopy

Nodules from Cd-treated and untreated plants of wild-type SGE and the mutant SGECd^t^ were harvested and placed directly into the fixative. The nodules were fixed in 3% paraformaldehyde and 0.1% glutaraldehyde (Sigma-Aldrich, St. Louis, MO, USA) in 0.01% PBS (2.48 g/L NaH_2_PO_4_, 21.36 g/L Na_2_HPO_4_, 87.66 g/L NaCl, pH 7.2). After fixation, the samples were post-fixed in 2% osmium tetroxide in a 0.1 M phosphate buffer for 2 h; each sample was then dehydrated, as described previously (Serova et al., 2018), and progressively infiltrated with Eponate 12 (Ted Pella Inc., Redding, CA, USA) at room temperature. Embedded samples were transferred to small plastic containers in fresh resin, following the manufacturer’s instructions, and polymerized at 60°C for 48 h.

For light microscopy, semi-thin sections (1-μm-thick) were cut on a Leica EM UC7 ultramicrotome (Leica Microsystems, Wetzlar, Germany). Sections were placed on glass slides SuperFrost (Menzel-Gläser, Thermo Fisher Scientific, Waltham, MA USA) and stained with methylene blue-azure II for 20 min at 70°C (Humphrey and Pittman, 1974) for later examination under a microscope Axio Imager.Z1 (Zeiss). Photos were taken using a digital video camera Axiocam 506 (Zeiss). For transmission electron microscopy, 90–100-nm-thick ultrathin sections were cut and counterstained as described by Serova et al. (2018). The nodule tissues were examined and photographed under a Tecnai G2 Spirit electron microscope (FEI, Eindhoven, the Netherlands) at 80 kV. Digital micrographs were taken with a MegaView G2 CCD camera (Olympus-SIS, Münster, Germany).

### Cadmium and Nutrient Contents in Plants

Dried plant shoots were ground into powder. To determine their Cd and nutrient—P, K, Ca, Mg, S, Fe, B, Mn, Co, Cu, Mo, Na, Ni, and Zn—contents, ground shoot samples were digested in a mixture of concentrated HNO_3_ and 38% H_2_O_2_ at 70°C using the DigiBlock digester (LabTech, Sorisole, Italy), and the content of each element measured by an inductively coupled plasma emission spectrometer ICPE-9000 (Shimadzu, Tokyo, Japan). Total nitrogen in shoot samples was determined using a Kjeltec 8200 Auto distillation unit (FOSS Analytical, Hillerød, Denmark). Statistical analysis of the data was performed using the STATISTICA v10 (StatSoft Inc., Tulsa, OK, USA). ANOVA analysis and Fisher’s least significant difference (LSD) test were used to evaluate differences between means.

## Results and Discussion

### Bacterial Strain Construction, Plasmid Stability, and Strain Tolerance to Cadmium

To create a symbiotic system capable of accumulating Cd, transgenic strains of *R. leguminosarum* bv. *viciae* 3841-PsMT1 and 3841-PsMT2 were first obtained. These strains carried the genes of plant metallothioneins *PsMT1* and *PsMT2* fused with the *nifH* gene promoter of *R. leguminosarum* bv. *viciae*. Encoding a small subunit of nitrogenase, *nifH* has a strong promoter providing the stable transcription of a gene under its control in micro-aerophilic conditions, which characterize the symbiotic nodule.

At first, we tested plasmid stability in the transgenic strains of *R. leguminosarum* bv. *viciae* 3841-PsMT1 and 3841-PsMT2. In the absence and presence of Cd, all the recovered clones of strains 3841-PsMT1 and 3841-PsMT2 were found to be resistant to kanamycin, indicating that the culturable nodule bacteria maintained 100% of pCAMBIA plasmids in nodules of the wild-type SGE and the mutant SGECd^t^.

In addition, these strains grew similarly on a liquid medium without Cd, as well as in the presence of 5 and 10 μm of CdCl_2_, indicating that treatment is not affecting bacteria viability.

In contrast to this study, in previous studies, rhizobial transgenic strains used in “symbiotic engineering” technology for phytoremediation of Cd-contaminated soils were created based on alien genes for the symbiotic system (Sriprang et al., 2002, 2003; Ike et al., 2007).

### Nodule Phenotype Analysis

**Figure 1:**
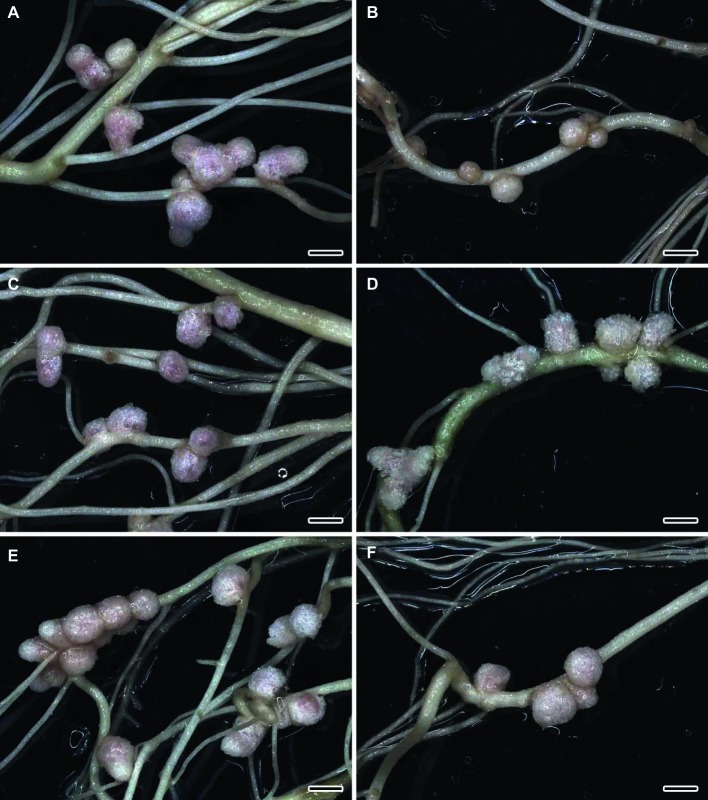
Nodulated main and lateral roots of wild-type SGE pea (*Pisum sativum*) plants untreated **(A,C,E)** and treated with cadmium chloride **(B,D,F)** at 28 days after inoculation with *Rhizobium leguminosarum* bv. *viciae* strain 3841 **(A,B)**, 3841-PsMT1 **(C,D)**, and 3841-PsMT2 **(E,F)**. Scale bar = 2 mm.

**Figure 2:**
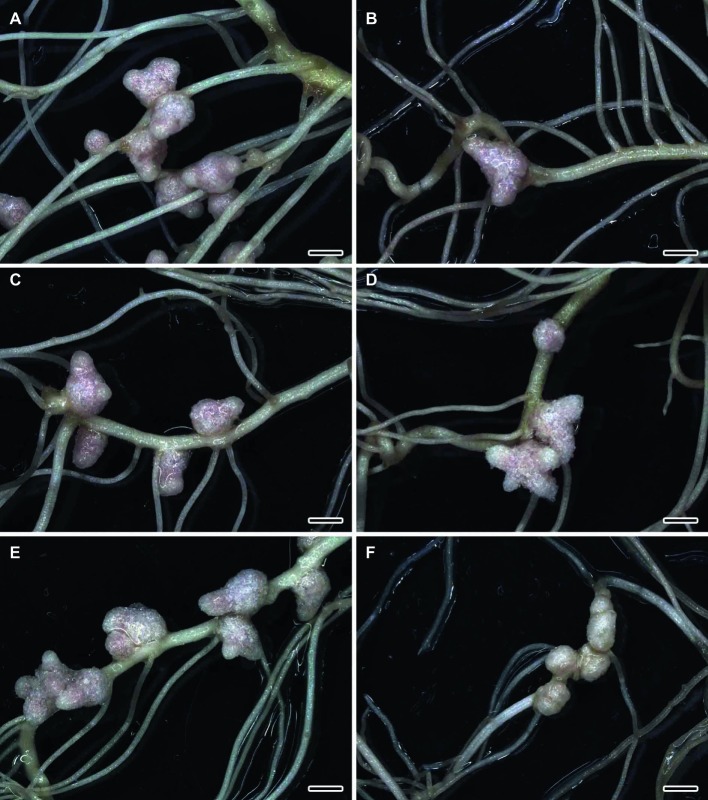
Nodulated main and lateral roots of the mutant SGECd^t^ pea (*Pisum sativum*) plants untreated **(A,C,E)** and treated with cadmium chloride **(B,D,F)** at 28 days after inoculation with *Rhizobium leguminosarum* bv. *viciae* 3841 **(A,B)**, 3841-PsMT1 **(C,D)**, and 3841-PsMT2 (**E,F**). Scale bar = 2 mm.

The formation of effective pink nodules was observed in the wild-type SGE inoculated with *R. leguminosarum* bv. *viciae* strains 3841, 3841-PsMT1, or 3841-PsMT2 (Figures 1A,C,E). Treatment with 0.5 μM CdCl_2_ resulted in the formation of several small pale nodules on wild-type plants inoculated with 3841 (Figure 1B); however, pink nodules developed on wild-type plants inoculated with transgenic strains 3841-PsMT1 and 3841-PsMT2 treated with Cd (Figures 1D,F). The mutant SGECd^t^ pea plants also formed pink nodules after inoculation with 3841, 3841-PsMT1, or 3841-PsMT2 (Figures 2A,C,E). The SGECd^t^ plants inoculated with either 3841 or 3841-PsMT1 still formed pink nodules after treatment with 0.5 μM CdCl_2_ (Figures 2B,D), while nodules that formed on 3841-PsMT2 were small and pale (Figure 2F).

The wild-type strain 3841 and transgenic strains 3841-PsMT1 and 3841-PsMT2 formed normal pink nodules in hydroponic solution without Cd also, as previously described for the strain CIAM 1026 (Tsyganov et al., 2004). Thus, the introduction of genetic constructions did not alter the ability of modified strains to undergo nodulation. In work by Tsyganov et al. (2005), the ability of the mutant SGECd^t^ to form nodules resistant to Cd stress upon inoculation with wild-type strain 3841, in contrast to the wild-type SGE, was demonstrated. Yet, surprisingly, transgenic strain 3841-PsMT2 formed pale nodules that may be indicative of their inefficiency.

### Expression Analysis

**Figure 3:**
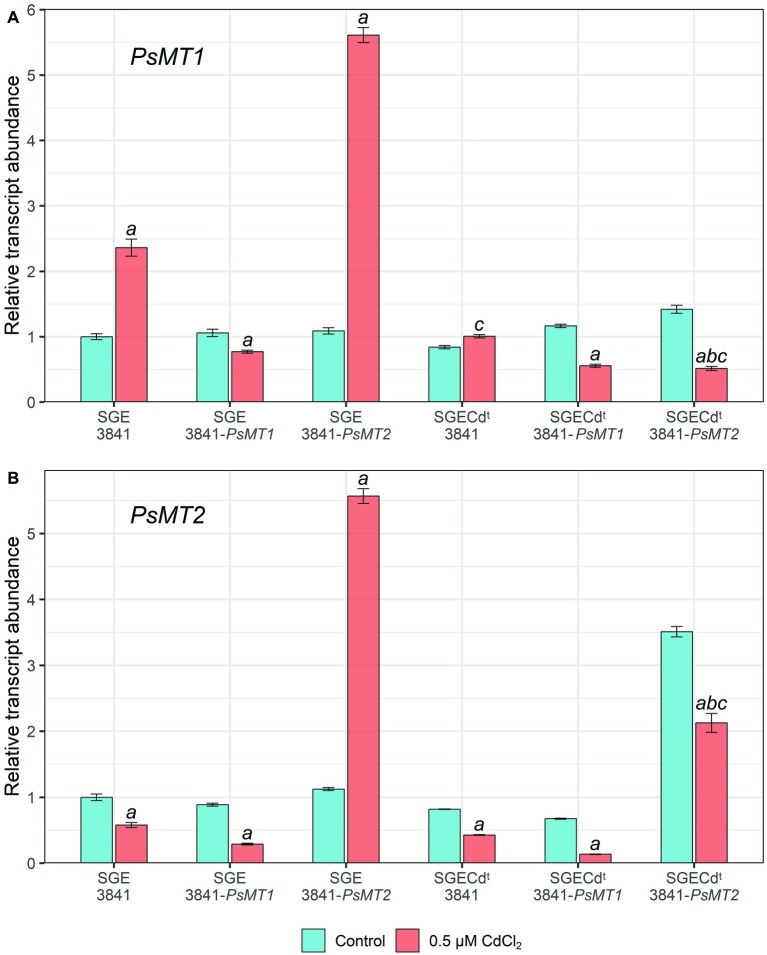
Expression of *PsMT1* **(A)** and *PsMT2* **(B)** genes in the nodules of pea (*Pisum sativum*) plants grown hydroponically without cadmium stress (control) and with the addition of 0.5 μM CdCl_2_. Letters indicate significant differences (two-way ANOVA, *p* ≤ 0.05): *a*, from the control; *b*, of the control SGECd^t^ from control wild-type plants inoculated with the same strain of *Rhizobium leguminosarum* bv. *viciae*; *c*, of treated SGECd^t^ from the treated wild type inoculated with the same strain of *R. leguminosarum* bv. *viciae*.

In wild-type nodules, *PsMT1* gene expression was significantly increased (2.4-fold) under the Cd treatment compared with untreated nodules when plants were inoculated with strain 3841 (Figure 3A). This gene’s expression level in the nodules of the SGECd^t^ inoculated with strain 3841 remained unchanged, being 2.3-fold lower than in wild-type nodules formed upon inoculation with the same strain (Figure 3A). On the contrary, the expression level of the *PsMT2* gene was reduced in nodules formed by strain 3841 on roots of both the wild type and mutant, which may indicate an insignificant role of this gene in detoxification of Cd in the nodules.

Paradoxically, *PsMT1* gene expression did not increase in nodules formed on roots of both the wild type and SGECd^t^ by strain 3841-PsMT1 carrying the insertion of this gene when compared to strains without this gene (Figure 3A). Perhaps this is caused by gene repression due to the introduction of additional copies of the *PsMT1* gene into the rhizobial strain.

In nodules of wild-type plants inoculated with strain 3841-PsMT2, the expression of both *PsMT1* and *PsMT2* genes during the Cd treatment increased 5-fold compared with the untreated control (Figure 3). This may indicate general regulatory mechanisms and the relationship between overexpression of the *PsMT1* and *PsMT2* genes in the pea nodules of the SGE line under the influence of Cd.

Yet the expression levels of *PsMT1* and *PsMT2* went unchanged or decreased in nodules formed by any tested strains on roots of the mutant SGECd^t^. This would correspond to a significantly lower activation of different responses to Cd in this mutant than in the wild type (Tsyganov et al., 2007).

The expression of different genes of plant metallothioneins demonstrates some organ and metal specificity (Joshi et al., 2016). Recently, it was shown that Cu application caused in tomatoes an increase in the expression of the *MT1* and *MT2* genes in the roots, leaves, and fruits. However, Pb treatment led to increased expression of these genes only in leaves. Moreover, with an increase in Pb concentration, the level of *MT1* gene expression decreased (Kisa et al., 2017). Thus, the regulatory mechanism of gene expression of metallothionenins is still far from elucidation.

Previously, we compared the expression levels of genes encoding key Cd detoxification enzymes in 4-week-old nodules formed by strain *R. leguminosarum* bv. *viciae* 3841 in wild-type SGE and mutant SGECd^t^ exposed to 0.5 μM CdCl_2_ (Kulayeva and Tsyganov, 2015). These genes were *PsGSH1* (γ-glutamylcysteine synthetase), *PsGSHS* (glutathione synthetase), *PshGSHS* (homoglutathione synthetase), and *PsPCS* (phytochelatin synthase). The expression of *PsGSH1* was not altered under Cd exposure in the wild-type nodules, and it was decreased in the mutant SGECd^t^. Similarly, expression of *PsGSHS* was reduced in the mutant nodules under the Cd exposure but it increased in wild-type nodules. In contrast to the first two genes, the levels of expression of *PshGSHS* and *PsPCS* were elevated in nodules of both the wild type and the mutant under Cd exposure. However, expression of *PshGSHS* and *PsPCS* increased to a large extent in wild-type nodules, which indicated that (homo)glutathione and phytochelatins are unlikely involved in the elevated Cd tolerance of mutant nodules (Kulayeva and Tsyganov, 2015).

### Nodule Histological and Ultrastructural Organization

#### Histological and Ultrastructural Organization of Cadmium-Treated and Untreated Nodules of the Wild-Type SGE and Mutant SGECd^t^ Inoculated With *R. leguminosarum* bv. *viciae* Strain 3841

**Figure 4:**
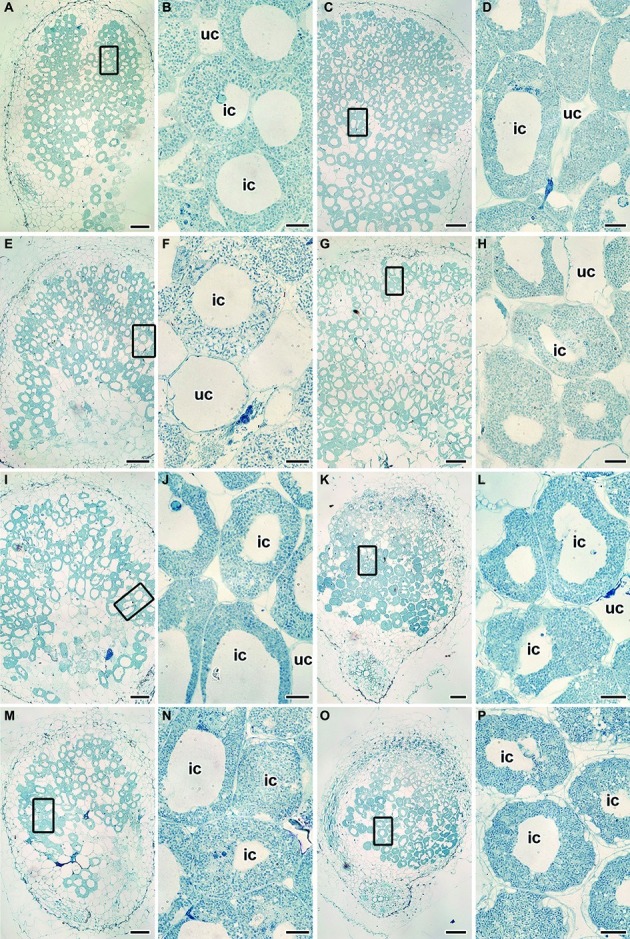
Histological organization of the untreated **(A–D)** and the cadmium-treated **(E–P)** nodules of wild-type SGE **(A,B,E,F,I,J,M,N)** and mutant SGECd^t^ **(C,D,G,H,K,L,O,P)** inoculated with *Rhizobium leguminosarum* bv. *viciae* strain 3841 **(A–H)**, 3841-MT1 **(I–L)** and 3841-MT2 (**M–P**). ic, infected cell; uc, uninfected cell. **(A,C,E,G,I,K,M,O)** Histological organization; **(B,D,F,H,J,L,N,P)** high magnification of the boxed area in **(A,C,E,G,I,K,M,O)**. Scale bar **(A,C,E,G,I,K,M,O)** = 200 μm, **(B,D,F,H,J,L,N,P)** = 20 μm.

Wild-type SGE (Figures 4A,B) and mutant SGECd^t^ (Figures 4C,D) plants inoculated with *R. leguminosarum* bv. *viciae* strain 3841 without Cd exposure formed indeterminate nodules having a typical histological organization (Tsyganov et al., 1998). Ultrastructural organization of the wild-type SGE (Supplementary Figure S1) and mutant SGECd^t^ (data not shown) was also similar. In the nitrogen fixation zone, each infected cell contained symbiosomes with a single differentiated pleiomorphic bacteroid surrounded by a symbiosome membrane (Supplementary Figure S1A). In the infection zone, juvenile bacteroids were observed in the narrow layer of cytoplasm (Supplementary Figure S1B).

**Figure 5:**
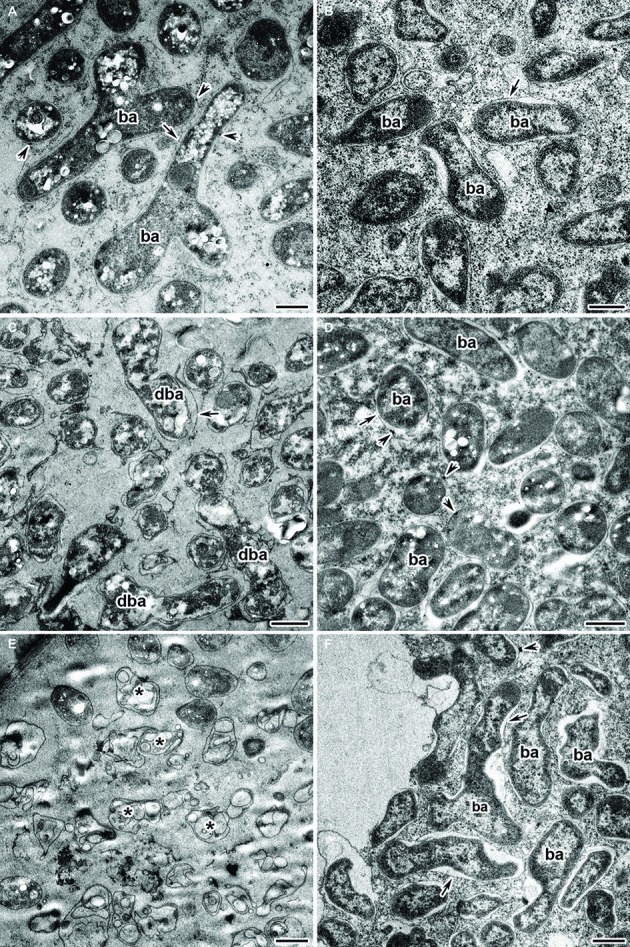
Ultrastructural organization of the cadmium-treated nodules of wild-type SGE **(A,C,E)** and mutant SGECd^t^ **(B,D,F)** inoculated with *Rhizobium leguminosarum* bv. *viciae* strain 3841. ba, bacteroid; dba, degrading bacteroid; arrows indicate the symbiosome membrane; arrowheads indicate the degradation of symbiosome membrane; asterisk used to indicate “ghost” bacteroids. **(A)** Mature bacteroids with polyhydroxybutyrate accumulation, **(B)** normal bacteroids, **(C)** degrading bacteroids, **(D)** bacteroids with polyhydroxybutyrate accumulation, **(E)** “ghost” bacteroids, **(F)** abnormal bacteroids. Scale bar = 500 nm.

Under the Cd treatment, most of the wild-type nodules showed certain symptoms of cytological damage in the nitrogen fixation zone such as cytoplasm clearing (Figures 4E,F), when compared with untreated nodules (Figures 4A,B) and Cd-treated nodules of the mutant SGECd^t^ (Figures 4G,H); in addition, an enlarged senescence zone was observed (Figure 4E). Nevertheless, some nodules were less affected while others incurred severe structural damage. Electron microscopy revealed the following abnormalities in the Cd-treated nodules of the wild type: polyhydroxybutyrate (PHB) accumulation in the mature bacteroids and destruction of the symbiosome membrane, with lipid peroxidation in the form of electron-dense formations (Figure 5A); some infected cells exhibited reduced cytoplasm with clear symptoms of plasmolysis (Figure 5C); organelles other than mitochondria and degrading bacteroids were hardly seen; finally, infected cells with destroyed cytoplasm “ghosts” of the bacteroids appeared (Figure 5E).

Unlike for the wild type, in the Cd-treated nodules of the mutant SGECd^t^ exposure to Cd caused less pronounced changes in their ultrastructural organization (Figures 5B,D,F). Most of the infected cells showed unchanged bacteroids and a cytoplasm with intact organelles present. In some infected cells, bacteroids with slight PHB accumulation and lipid peroxidation of the symbiosome membranes (Figure 5D) were detected. The more pronounced damage from Cd exposure manifested as an expansion of peribacteroid spaces and an irregular shape of the bacteroids (Figure 5F).

#### Histological and Ultrastructural Organization of Cadmium-Treated and Untreated Nodules of the Wild-Type SGE and Mutant SGECd^t^ Inoculated With *R. leguminosarum* bv. *viciae* Strain 3841-PsMT1

Wild-type SGE and mutant SGECd^t^ plants inoculated with *R. leguminosarum* bv. *viciae* strain 3841-PsMT1 without exposure to Cd formed nodules having a typical histological and ultrastructural organization (data not shown). Cd-treated nodules of the wild type and mutant SGECd^t^ formed by the strain 3841-PsMT1 did not cause any marked alterations to their histological organization when compared with nodules not receiving the Cd treatment (Figures 4I–L).

**Figure 6:**
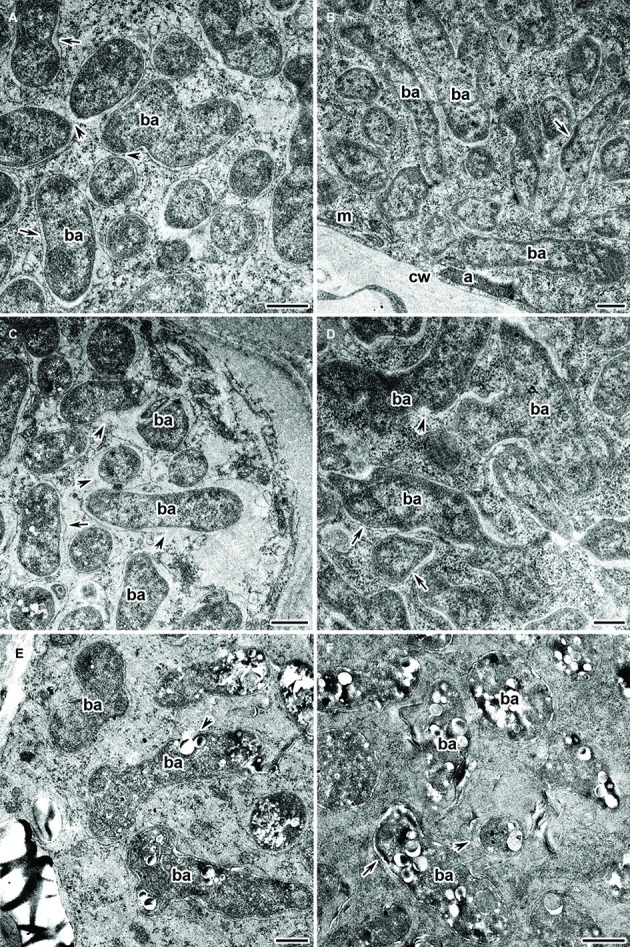
Ultrastructural organization of the cadmium-treated nodules of wild-type SGE **(A,C,E)** and mutant SGECd^t^ **(B,D,F)** inoculated with *Rhizobium leguminosarum* bv. *viciae* strain 3841-MT1. ba, bacteroid; cw, cell wall; a, amyloplast; m, mitochondrion; arrows indicate the symbiosome membrane; arrowheads indicate the degradation of symbiosome membrane. **(A,B)** Mature bacteroids, **(C)** symbiosomes with partial destruction of symbiosome membranes and cytoplasm swelling, **(D)** abnormal bacteroids, **(E,F)** mature bacteroids with polyhydroxybutyrate accumulation. Scale bar = 500 nm.

Electron microscopy revealed that the main part of infected cells in the nitrogen fixation zone of Cd-treated nodules had a similar ultrastructure in both the wild type and mutant SGECd^t^. Infected cells contained well-developed bacteroids (Figures 6A,B) and normal infection threads (data not shown). The degenerative changes of infected cells were limited to the presence of an expanded peribacteroid space (Figure 6C), irregular shape of bacteroids (Figure 6D), damaged symbiosome membranes with lipid peroxidation (Figures 6A,C), and PHB accumulation (Figures 6E,F). In the rare infected cells, initial symptoms of cytoplasm swelling and clearing (Figure 6C), as well as organelle damage, could be observed (Figures 6E,F).

#### Histological and Ultrastructural Organization of Cadmium-Treated and Untreated Nodules of Wild-Type SGE and Mutant SGECd^t^ Inoculated With *R. leguminosarum* bv. *viciae* Strain 3841-PsMT2

Nodules of wild-type SGE and mutant SGECd^t^ plants formed by *R. leguminosarum* bv. *viciae* strain 3841-PsMT2 without exposure to Cd showed a typical histological and ultrastructural organization (data not shown). For both the wild type and mutant SGECd^t^ inoculated with strain 3841-PsMT2, the Cd treatment did not cause the significant alterations to their nodule histological organization (Figures 4M–P). By contrast, in the Cd-treated nodules of the mutant SGECd^t^ an intensive accumulation of starch was evident (Figures 4O,P).

**Figure 7:**
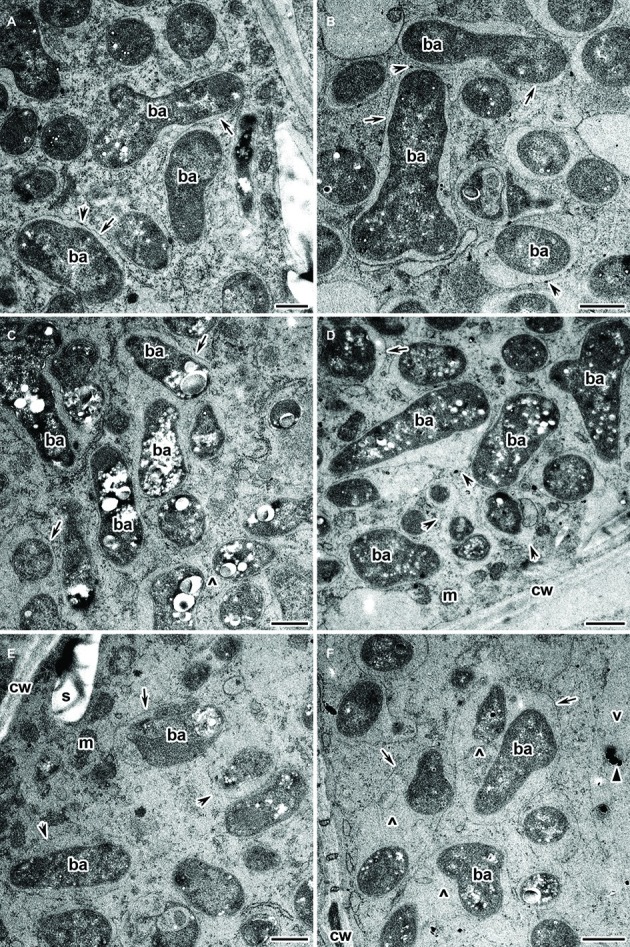
Ultrastructural organization of the cadmium-treated nodules of wild-type SGE **(A,C,E)** and mutant SGECd^t^ **(B,D,F)** inoculated with *Rhizobium leguminosarum* bv. *viciae* strain 3841-MT2. ba, bacteroid; cw, cell wall; m, mitochondrion; v, vacuole; s, starch granule; ^, “multiple” symbiosome, formed as a result of symbiosome fusion and containing several bacteroids; arrows indicate the symbiosome membrane; arrowheads indicate the degradation of symbiosome membrane; triangle indicates electron dense crystals in the vacuole. **(A,B)** Mature bacteroids, **(C,D)** mature bacteroids with polyhydroxybutyrate accumulation, **(E,F)** symbiosomes with partial destruction of symbiosome membranes and cytoplasm swelling. Scale bar = 500 nm.

Infected cells in the nitrogen fixation zone of both the wild type and mutant SGECd^t^ nodules under Cd exposure did not differ in their ultrastructural organization. All infected cells contained mature Y-shaped bacteroids (Figures 7A,B), and apparently normal infection threads (data not shown). The presence of an expanded peribacteroid space (Figures 7B–D), symbiosome membrane destruction (Figures 7D,E), lipid peroxidation of symbiosome membranes (Figures 7A,B), and PHB accumulation (Figures 7C,D) were all observed in most of the infected cells of the Cd-treated nodules of both plant genotypes. Some infected cells did show a cleared cytoplasm with initial symptoms of plasmolysis (Figures 7E,F). In these cells, bacteroids were released into the vacuole (data not shown), and symbiosomes harbored several bacteroids as a result of symbiosome fusion (Figures 7C,F).

#### Depositions in the Vacuole in the Infected Cells of Cadmium-Treated Nodules of Wild-Type SGE and Mutant SGECd^t^ Inoculated With *R. leguminosarum* bv. *viciae* Strains 3841, 3841-MT1, and 3841-MT2

An exciting feature revealed by the experiment’s Cd treatment were depositions, in the form of electron-dense crystals, in the vacuoles of infected cells in nodules of both plant genotypes when inoculated with *R. leguminosarum* bv. *viciae* strains 3841, 3841-MT1, and 3841-MT2 (Supplementary Figure S2). These crystals were never found in both genotypes regardless of the strain used when unexposed to Cd (Supplementary Figure S1B). In nodules of the wild-type SGE formed by 3841, such structures in the vacuole were detected in senescent cells with “ghosts” of bacteroids and bacteria released from destroyed infection threads (Supplementary Figure S2A). In the nodules of wild-type SGE and mutant SGECd^t^ plants inoculated with 3841-PsMT1, small electron-dense crystals were observed in conglomerates of destroyed bacteroids and other cell debris (Supplementary Figures S2C,D). However, in the nodules of both plant genotypes formed by the strain 3841-MT2, electron-dense crystals in the vacuole occurred in the infected cells with a safe ultrastructural organization (Supplementary Figure S2E), as well as in those with initial symptoms of cytoplasm plasmolysis (Supplementary Figure S2F).

Recently, the effects of 24-h exposure to 100 μM and 1 mM CdCl_2_ on the histological and ultrastructural organization of symbiotic nodules of the wild-type SGE and mutant SGECd^t^ plants inoculated with strain 3841 were studied (Tsyganova et al., 2019). In those wild-type nodules, the Cd treatment caused the formation of an enlarged peribacteroid space in the symbiosomes, along with destruction of the symbiosome membrane and symbiosome fusion, which led to the formation of symbiosomes containing several bacteroids; however, when treated with 1 mM CdCl_2_, the infected cells underwent cytoplasm clearing and contained destroyed bacteroids. These nodule abnormalities were less pronounced in the mutant SGECd^t^ (Tsyganova et al., 2019). In *M. sativa* nodules exposed to Cd, evidence for some damaged cells and plasmolysis and destroyed bacteroids were reported, but in some nodules Cd caused less severe degradation, and those infected cells contained bacteroids with an enlarged peribacteroid space and symbiosomes with several bacteroids (Shvaleva et al., 2010). Thus, the observed abnormalities in nodule functioning in our current study are similar to those described before. However, here we also discerned PHB accumulation in the bacteroids. Other work with pea plants demonstrated that PHB accumulation occurs in undifferentiated rhizobia in the infection threads of its nodules but this was absent in bacteroids (Lodwig et al., 2005). By contrast, a recent study showed that PHB accumulation is not restricted to bacteria *per se*, as an analysis of genome sequences identified functional PHB synthase (type III, PhaE PhaC2) in bacteroids (Terpolilli et al., 2016). Moreover, PHB is required for nitrogen fixation by *Azorhizobium caulinodans* in its free-living state and during symbiosis with *Sesbania rostrata* Bremek. & Oberm. (Mandon et al., 1998), but its synthesis may be blocked in bacteroids of other legume species (Cevallos et al., 1996; Aneja and Charles, 1999; Lodwig et al., 2005; Quelas et al., 2013). In other bacteria, apparently PHB may be produced in response to physiological stress. For example, genetic analysis using a phbC mutant strain of *Pseudomonas fluorescens* with host plants revealed that PHB could function as a beneficial microbial compound, one synthesized as the plant adjusts to mitigate cold stress (Stritzler et al., 2018).

We discovered depositions of electron-dense crystals in the vacuoles of infected cells in nodules of both wild type and mutant SGECd^t^ pea plants formed, by any of the tested strains, under exposure to Cd. However, the nature of these depositions is unclear. Previously, it was shown that Cd is mainly adsorbed by cell walls of the outer cortex and, to a lesser extent, by walls of cells from the infection zone (Sánchez-Pardo et al., 2013). Further studies are necessary to elucidate the nature of these depositions and their long-term effects on the plants.

In the recombinant strains 3841-PsMT1 and 3841-PsMT2 with respective heterologous expression of genes *PsMT1* and *PsMT2* encoding metallothioneins, the negative influence of Cd on nodule development was less pronounced. This result may indicate that metallothioneins have a positive effect on the histological and ultrastructural organization of pea nodules when formed under exposure to Cd in the local environment. Yet, in this respect, strain 3841-PsMT1 clearly had a stronger effect. A more severe alteration of nodule ultrastructure in nodules formed with 3841-PsMT2 on plants of SGECd^t^ was likely driven by increased Cd accumulation in these nodules. In this variant, at least, the highest Cd content in shoots was observed (see below), and its nodules looked pale while its cells contained many starch granules, a key trait of nodule ineffectiveness (Forrest et al., 1991; Serova et al., 2018).

### Cadmium and Nutrient Contents in Plants

**Figure 8:**
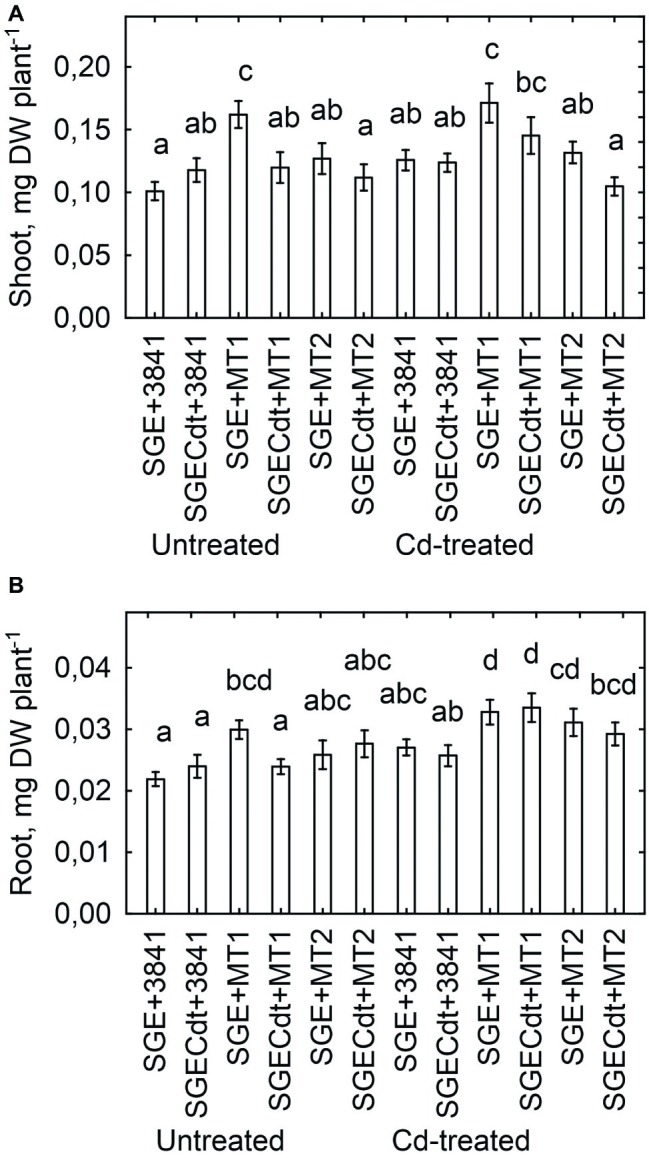
Shoot **(A)** and root **(B)** biomass of pea (*Pisum sativum*) plant genotypes SGE and SGECd^t^ grown in nutrient solution and inoculated with *Rhizobium leguminosarum* bv. *viciae* strains 3841, 3841-PsMT1 (marked as MT1) or 3841-PsMT2 (marked as MT2), respectively. Vertical bars show standard errors of the means. Different letters show significant differences between the treatments (least significant difference test, *p* < 0.05, *n* = 5). DW denotes dry weight.

Inoculation with strain 3841-PsMT1 increased the shoot (Figure 8A) and root (Figure 8B) biomass of both untreated and Cd-treated wild-type plants, when compared with those inoculated with strain 3841. But a positive response of mutant SGECd^t^ to strain 3841-PsMT1 relative to strain 3841 was only found to be significant for roots of Cd-treated plants (Figure 8B). Indeed, plants inoculated with strains 3841 and 3841-PsMT2 had a similar biomass for both pea genotypes when grown in the absence or presence of Cd in the nutrient solution (Figure 8), and the growth response of the wild type and mutant SGECd^t^ to the Cd treatment was also similar. In our other work, treatments consisting of 3 μM CdCl_2_ (Tsyganov et al., 2007) or 4 μM CdCl_2_ (Belimov et al., 2015a) revealed significant differences between the wild type and mutant SGECd^t^ plants in terms of their biomass production, with the mutant exhibiting greater tolerance to Cd toxicity. But the response of both pea genotypes to 0.5 μM CdCl_2_ was insignificant, as they had a similar shoot biomass (Kulayeva and Tsyganov, 2015; Belimov et al., 2015b). It is quite likely then that negligible adverse effects of Cd on plant growth and genotypic differences in our current experiment were caused by relatively low Cd concentration in the nutrient solution. Nonetheless, inserting the *PsMT1* gene into nodule bacterium 3841 led to an increased root biomass in both pea genotypes and a greater shoot biomass of SGE grown in the Cd-supplemented solution (Figure 8). Yet, strain 3841 with the *PsMT2* gene could only increase the root biomass of Cd-treated SGECd^t^.

**Figure 9:**
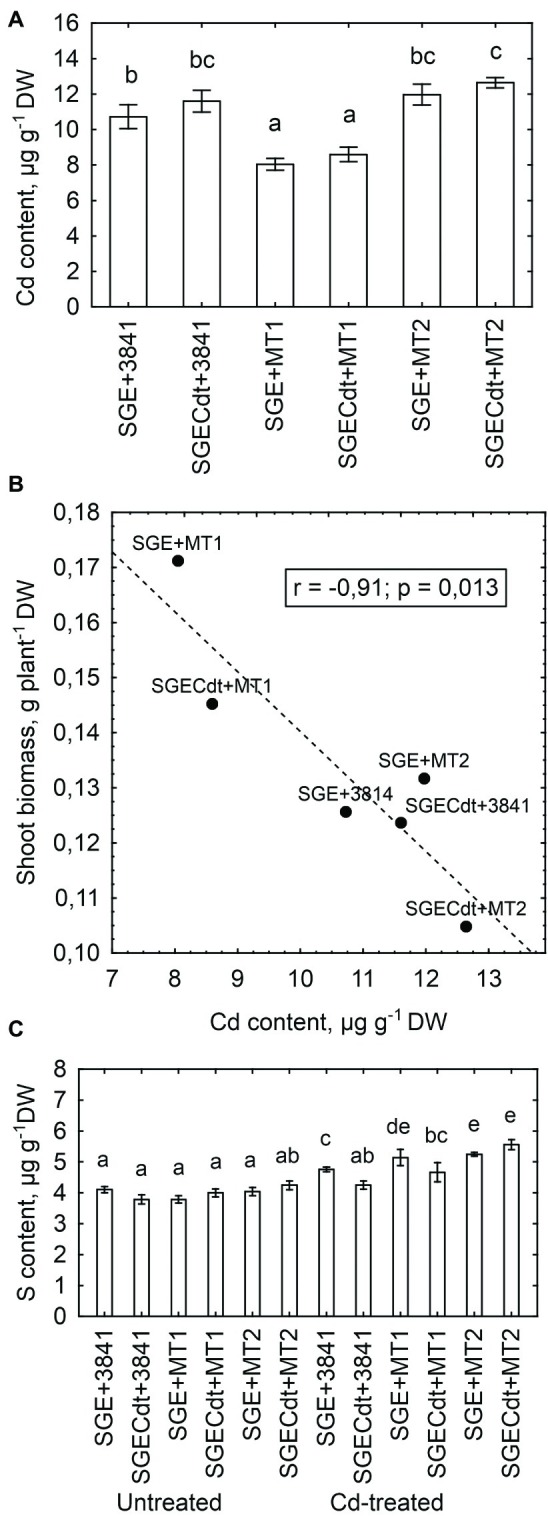
Shoot Cd content in the cadmium-treated pea (*Pisum sativum*) plants **(A)**, and linear regressions between shoot biomass and shoot Cd content **(B)** and shoot S content **(C)** in pea genotypes SGE and SGECd^t^ grown in nutrient solution and inoculated with *Rhizobium leguminosarum* bv. *viciae* strains 3841, 3841-PsMT1 (marked as MT1) or 3841-PsMT2 (marked as MT2), respectively. Vertical bars show standard errors of the means. Different letters show significant differences between the treatments (least significant difference test, *p*<0.05, *n* = 5). DW denotes dry weight. Fitted linear regression on B is shown by the dashed line.

Strain 3841-PsMT1 decreased the Cd content of shoots in both pea genotypes, whereas strain 3841-PsMT2 had no significant effect upon shoot Cd content (Figure 9A). The maximum value for Cd content occurred in shoots of the mutant SGECd^t^ inoculated with 3841-PsMT2, which significantly differed from that of the wild type inoculated with the parental strain 3841. Although there were no significant genotypic differences within coincident inoculation treatments, a tendency for greater Cd content in the mutant SGECd^t^ should be mentioned. Previously, under toxic Cd concentrations 3 μM and 4 μM of CdCl_2_, the mutant SGECd^t^ accumulated Cd more actively than did its wild-type counterpart (Tsyganov et al., 2007; Belimov et al., 2015a). Those findings suggested the plant-microbe combination based on the plant genotype SGECd^t^, with its stronger Cd accumulation capacity, and the genetically modified micro-symbiont carrying *PsMT2* gene would be the most efficient for increasing tissue Cd concentrations. However, in our study here, this combination did not lead to increased accumulation of Cd by shoots because of the relatively low shoot biomass of the mutant SGECd^t^ (Figure 8A). Moreover, there was a negative correlation (*r* = −0.91, *p* = 0.013, *n* = 6) between shoot biomass of Cd-treated plants and their shoot Cd content (Figure 9B), suggesting that Cd accumulation by the plants—particularly the mutant SGECd^t^—inoculated with 3841-PsMT2 was sufficient to contribute to growth inhibition vis-à-vis those inoculated with strain 3841-PsMT1. The reasons underpinning the opposing effects of 3841-PsMT1 and 3841-PsMT2 on Cd content merit more detailed study. We propose that metallothioneins produced by the bacterial strains differ in their capability for release from bacterial cells, thereby bounding Cd and/or transporting this metal throughout plant tissues, from its root to shoot parts aboveground.

Metallothioneins bind heavy metals through the thiol group of its cysteine residues (Joshi et al., 2016); hence, sulfur (S) is a crucial element for their functioning. As a rule, in our experiment the Cd-treated plants inoculated with strains 3841-PsMT1 or 3841-PsMT2 had a higher shoot S content than those inoculated with strain 3841, in addition to the Cd-untreated plants (Figure 9C). However, Cd and S contents of shoots from Cd-treated plants were not significantly correlated (*r* = +0.24, *p* = 0.21, *n* = 30). Therefore, it remains unclear whether metallothioneins participate in a crucial way in Cd translocation from root to shoot. Further, careful measurements of metallothioneins in pea shoots are necessary to robustly test this hypothesis. Nonetheless, the differences we found could be explained by existing differences in organ/tissue-specific expression patterns of the used genes. Indeed, we know that type 1 metallothioneins are mainly expressed in roots, types 2 and 3 in leaves, and type 4 in seeds (Joshi et al., 2016).

Insertion of *PsMT1* and *PsMT2* genes into rhizobia affected the content of nutrient elements in the experimental pea shoots (Supplementary Table S2). Compared with strain 3841, inoculation with 3841-PsMT1 decreased the Cu, K, Mo, and P content of shoots in Cd-untreated SGE plants and the B, Fe, K, and Zn content of shoots in Cd-treated SGE plants. The effect of 3841-PsMT1 on nutrients in SGECd^t^ shoots was generally insignificant with a few exceptions: an increased Ca content and decreased N and Zn content of Cd-treated plants. Generally, the N content was little affected by the inoculations with 3841-PsMT1 or 3841-PsMT2, and the only significant genotypic difference (one of 13%) was detected between the Cd-treated wild type and mutant plants inoculated with 3841-PsMT1. Inoculation with 3841-PsMT2 decreased the K and Mo contents of shoots in Cd-untreated SGE plants and the Co, Fe, and Na content in shoots of Cd-treated SGE plants. However, the response of the mutant SGECd^t^ to inoculation with 3841-PsMT2 differed significantly from SGE. In particular, the B, Mg, and Mn contents of Cd-untreated plants and B, Co, Cu, K, Mg, Mn, Mo, Na, and Zn contents of Cd-treated plants were all increased. Moreover, differences between Cd-treated SGE and SGECd^t^ in their tissues’ element contents were as a rule significant after inoculation with 3841-PsMT2. These results suggest that strain 3841-PsMT2 improved nutrient uptake by plants, particularly in the case of the Cd-treated SGECd^t^ mutant. It is known that metallothioneins can bind various elements (Joshi et al., 2016), so it its plausible that uptake of some nutrients was accompanied by 3841-PsMT2. Analysis of averaged values for each inoculation treatment showed that the Cd treatment had a negative effect on B, Cu, Na, Ni, and P contents, but it increased the Mg and Mn contents of SGE and/or SGECd^t^ plants’ shoots (Supplementary Table S2). These findings are in line with our previous reports showing Cd affected element composition of the studied pea genotypes as well as better uptake of nutrients by SGECd^t^ in the presence of toxic Cd concentrations (Tsyganov et al., 2007; Belimov et al., 2016).

## Conclusion

To our best knowledge, this work is the first example of a legume-rhizobial system developed for Cd phytoremediation in which both partners are genetically modified. Two strains, 3841-PsMT1 and 3841-PsMT2, were constructed and used as rhizobial inoculants of wild-type pea plants and a mutant with a known increased tolerance to Cd. Both strains differ in their effects on plant growth with and without Cd treatment, Cd and nutrient contents in host plant shoots, as well as their nodule histological and ultrastructural organization. The observed differences can be explained by different roles or different organ/tissue expression patterns of the used genes. Positive effects on plant biomass were noticed when the wild-type plants were inoculated with strain 3841-PsMT1. Importantly, this strain also decreased the Cd content of shoots in both pea genotypes and their nodules were able to maintain their organization under exposure to Cd. These features make the 3841-PsMT1 strain an interesting one for inoculating pea plants to increase their biomass and to decrease the Cd concentration in plant tissues. The other strain, 3841-PsMT2, especially in combination with the pea Cd-tolerant mutant, accumulated the highest Cd level among other variants, making it an attractive candidate for the phytoremediation of Cd-contaminated soil. Still, further experiments are needed to elucidate the required conditions for its possible practical applications.

## Data Availability Statement

All datasets generated for this study are included in the article/Supplementary Material.

## Author Contributions

IT designed the experiments. VK and EC obtained the transgenic strains. VK analyzed the plasmid stability. AG and AB analyzed strain tolerance to Cd. AG analyzed growth parameters. TS performed the nodule phenotype analysis. ES, AT, AK, and AG performed the light and electron microscopy. AB performed the elemental analysis. KI, OK, TS, and PK conducted the real-time PCR analysis. AT, AB, and VT analyzed the data and wrote the paper.

### Conflict of Interest

The authors declare that the research was conducted in the absence of any commercial or financial relationships that could be construed as a potential conflict of interest.

## Footnotes

^1^https://www.graphpad.com/scientific-software/prism/
