# Supplementary material for: Efficacy of a Plant-Microbe System: Pisum sativum (L.) Cadmium-Tolerant Mutant and Rhizobium leguminosarum Strains, Expressing Pea Metallothionein Genes PsMT1 and PsMT2, for Cadmium Phytoremediation
Source: Front Microbiol. 2020 Jan 29;11:15. doi: 10.3389/fmicb.2020.00015 (PMC7000653; doi:10.3389/fmicb.2020.00015)

Supplementary Material

Efficacy of a Plant-Microbe System: *Pisum sativum* (L.) Cadmium-Tolerant Mutant and *Rhizobium leguminosarum* Strains, Expressing Pea Metallothionein Genes *PsMT1* and *PsMT2*, for Cadmium Phytoremediation

Viktor E. Tsyganov^1,2*^, Anna V. Tsyganova^1^, Artemii P. Gorshkov^1^, Elena V. Seliverstova^1,3^, Viktoria E. Kim^1^, Elena P. Chizhevskaya^1^, Andrey A. Belimov^1^, Tatiana A. Serova^1^, Kira A. Ivanova^1^, Olga A. Kulaeva^1^, Pyotr G. Kusakin^1^, Anna B. Kitaeva^1^, Igor A. Tikhonovich^1,4^

^1^All-Russia Research Institute for Agricultural Microbiology, Pushkin 8, Saint-Petersburg, Russia

^2^Saint Petersburg Scientific Center RAS, Universitetskaya embankment 5, 199034, Russia

^3^Sechenov Institute of Evolutionary Physiology and Biochemistry of the Russian Academy of Sciences, Toreza prospect 44, Saint Petersburg, 194223, Russia

^4^Saint Petersburg State University, Department of Genetics and Biotechnology, Saint Petersburg, Russia

*** Correspondence:**Viktor E. Tsyganov
tsyganov@arriam.spb.ru

Supplemental Table 1. Primers used in this study.

| **Primer name** | **Sequence (5’–3’)** |
| --- | --- |
| MT1-F | ATGTCTGGATGTGGTTGTGGA |
| MT1-R | GCCTCCAATATCTCTGCTTCA |
| MT2-F | ATGTCTTGCTGTGGTGGAAACT |
| MT2-R | ATCCTGCCACTAAACGGGG |
| nifH-F | GGATCCCGTCGTTGCCTGCTG |
| nifH-R | GTTTGGCGTTCCTTCATGTGTTC |
| PsMT-1F | ATGTCTGGATGTGGTTGTGGAAG |
| PsMT-1R | TCATTTGCAGTTGCAAGGGTCA |
| PsMT-2F | TGGAAACTGTGGTTGCGGTACTAG |
| PsMT-2R | TCCACATTTGCAGCCATCATTCTC |

Supplemental Table 2. Content of nutrient elements in shoots of pea genotypes SGE and SGECd^t^ grown in nutrient solution and inoculated with *R. leguminosarum* bv. *viciae* strains 3841, 3841-PsMT1 or 3841-PsMT2, respectively.

| Treatments | B,  ng g^-1^ DW | Ca,  mg g^-1^ DW | Co,  µg g^-1^ DW | Cu,  µg g^-1^ DW | Fe,  µg g^-1^ DW | K,  mg g^-1^ DW | Mg,  mg g^-1^ DW | Mn,  µg g^-1^ DW | Mo,  µg g^-1^ DW | N,  mg g^-1^ DW | Na,  µg g^-1^ DW | Ni,  µg g^-1^ DW | P,  mg g^-1^ DW | Zn,  µg g^-1^ DW |
| --- | --- | --- | --- | --- | --- | --- | --- | --- | --- | --- | --- | --- | --- | --- |
|  | **Untreated plants** | | | | | | | | | | | | | |
| SGE + 3841 | 28 ± 0.8^cd^ | 8,0 ± 0.2^abc^ | 4,4 ± 0.1^ab^ | 84 ± 2^d^ | 73 ± 8^cd^ | 30 ± 0.2^cd^ | 5,1 ± 0.1^a^ | 64 ± 1^a^ | 46 ± 2^b^ | 18.1 ± 0.6^abc^ | 288 ± 12^d^ | 3,6 ± 0.1^c^ | 70 ± 2^c^ | 107 ± 4^cd^ |
| SGECd^t^ + 3841 | 27 ± 0.8^cd^ | 8,6 ± 0.2^c^ | 4,4 ± 0.1^ab^ | 73 ± 3^bc^ | 68 ± 5^bc^ | 30 ± 0.6^cd^ | 5,1 ± 0.2^a^ | 67 ± 3^a^ | 37 ± 1^a^ | 17.8 ± 0.5^abc^ | 262 ± 20^cd^ | 3,5 ± 0.1^bc^ | 58 ± 3^b^ | 102 ± 2^c^ |
| SGE + 3841-PsMT1 | 26 ± 0.8^bc^ | 8,3 ± 0.2^bc^ | 4,4 ± 0.1^ab^ | 72 ± 2^ab^ | 72 ± 5^cd^ | 28 ± 0.7^b^ | 5,2 ± 0.1^a^ | 66 ± 2^a^ | 32 ± 2^a^ | 17.4 ± 0.7^ab^ | 248 ± 12^bcd^ | 3,4 ± 0.1^bc^ | 59 ± 2^b^ | 86 ± 3^bc^ |
| SGECd^t^ + 3841-PsMT1 | 26 ± 0.6^bc^ | 8,4 ± 0.3^bc^ | 4,2 ± 0.1^ab^ | 72 ± 3^ab^ | 69 ± 8^c^ | 28 ± 0.9^b^ | 5,0 ± 0.2^a^ | 65 ± 2^a^ | 38 ± 3^a^ | 17.7 ± 0.9^abc^ | 246 ± 19^bcd^ | 3,2 ± 0.1^bc^ | 58 ± 3^b^ | 91 ± 3^bc^ |
| SGE + 3841-PsMT2 | 33 ± 1.6^e^ | 7,3 ± 0.4^ab^ | 4,4 ± 0.1^a^ | 79 ± 1^bcd^ | 58 ± 4^abc^ | 28 ± 0.3^b^ | 5,4 ± 0.1^ab^ | 68 ± 3^ab^ | 36 ± 2^a^ | 19.7 ± 0.7^bc^ | 273 ± 7^cd^ | 3,4 ± 0.1^bc^ | 62 ± 1^bc^ | 93 ± 3^bc^ |
| SGECd^t^ + 3841-PsMT2 | 36 ± 1.0^e^ | 7,3 ± 0.4^ab^ | 4,4 ± 0.2^ab^ | 75 ± 2^bc^ | 55 ± 7^abc^ | 29 ± 0.6^bc^ | 5,6 ± 0.3^bc^ | 83 ± 3^c^ | 37 ± 2^a^ | 19.0 ± 1.4^bc^ | 294 ± 32^d^ | 3,4 ± 0.3^bc^ | 59 ± 3^b^ | 96 ± 2^bc^ |
|  | **Plants treated with 0.5 µM CdCl_2_** | | | | | | | | | | | | | |
| SGE + 3841 | 27 ± 1.7^bcd^ | 7,5 ± 0.5^ab^ | 4,5 ± 0.1^bc^ | 75 ± 2^bcd^ | 86 ± 5^d^ | 31 ± 0.5^cd^ | 6,0 ± 0.2^cd^ | 105 ± 4^de^ | 36 ± 3^a^ | 19.8 ± 0.4^c^ | 252 ± 15^bcd^ | 3,3 ± 0.1^bc^ | 60 ± 2^b^ | 106 ± 7^cd^ |
| SGECd^t^ + 3841 | 24 ± 1.3^abc^ | 8,0 ± 0.5^a^ | 4,1 ± 0.1^ab^ | 70 ± 4^ab^ | 58 ± 3^abc^ | 27 ± 1.2^ab^ | 5,6 ± 0.2^bc^ | 101 ± 5^de^ | 34 ± 3^a^ | 19.9 ± 1.0^c^ | 171 ± 15^a^ | 2,7 ± 0.1^ab^ | 56 ± 4^ab^ | 97 ± 7^c^ |
| SGE + 3841-PsMT1 | 18 ± 1.1^a^ | 7,2 ± 0.5^a^ | 4,3 ± 0.2^ab^ | 65 ± 4^ab^ | 56 ± 5^abc^ | 28 ± 1.2^b^ | 6,1 ± 0.3^cd^ | 90 ± 6^cd^ | 38 ± 3^a^ | 18.6 ± 0.7^bc^ | 198 ± 15^ab^ | 2,8 ± 0.2^ab^ | 51 ± 4^ab^ | 73 ± 4^a^ |
| SGECd^t^ + 3841-PsMT1 | 23 ± 0.8^b^ | 8,3 ± 0.5^bc^ | 3,9 ± 0.2^b^ | 64 ± 3^a^ | 50 ± 3^a^ | 25 ± 0.8^a^ | 5,7 ± 0.3^bcd^ | 98 ± 6^de^ | 34 ± 4^a^ | 16.2 ± 0.3^a^ | 160 ± 12^a^ | 2,5 ± 0.1^a^ | 49 ± 3^a^ | 73 ± 4^a^ |
| SGE + 3841-PsMT2 | 24 ± 1.0^b^ | 8,1 ± 0.5^abc^ | 4,3 ± 0.1^a^ | 72 ± 2^b^ | 69 ± 5^c^ | 32 ± 0.7^d^ | 6,2 ± 0.2^de^ | 106 ± 7^e^ | 38 ± 1^a^ | 17.9 ± 0.9^abc^ | 155 ± 8^a^ | 2,8 ± 0.1^ab^ | 59 ± 2^b^ | 89 ± 3^bc^ |
| SGECd^t^ + 3841-PsMT2 | 30 ± 1.1^d^ | 7,6 ± 0.2^ab^ | 5,0 ± 0.3^c^ | 81 ± 5^cd^ | 76 ± 6^cd^ | 32 ± 0.8^d^ | 6,7 ± 0.2^e^ | 144 ± 7^f^ | 47 ± 3^b^ | 18.2 ± 0.7^abc^ | 230 ± 38^bc^ | 3,5 ± 0.3^bc^ | 65 ± 5^bc^ | 114 ± 4^d^ |
|  | **Average for all inoculation treatments** | | | | | | | | | | | | | |
| Untreated SGE | 29 ± 1.0^#^ | 7,9 ± 0.2* | 4,4 ± 0.1* | 78 ± 2^#^ | 68 ± 4* | 29 ± 0.4* | 5,2 ± 0.1* | 66 ± 1* | 38 ± 2* | 18.4 ± 0.4* | 270 ± 7^#^ | 3,5 ± 0.1^#^ | 64 ± 2^#^ | 95 ± 3* |
| Untreated SGECd^t^ | 30 ± 1.2^#^ | 8,1 ± 0.2* | 4,3 ± 0.1* | 73 ± 2*^#^ | 64 ± 4* | 29 ± 0.5* | 5,3 ± 0.1* | 72 ± 3* | 37 ± 1* | 18.1 ± 0.6* | 267 ± 14^#^ | 3,4 ± 0.1^#^ | 58 ± 2*^#^ | 96 ± 2* |
| Cd-treated SGE | 23 ± 1.2* | 7,6 ± 0.3* | 4,4 ± 0.1* | 71 ± 2* | 70 ± 4* | 30 ± 0.6* | 6,1 ± 0.1^#^ | 101 ± 4^#^ | 37 ± 1* | 18.8 ± 0.4* | 202 ± 13* | 3,0 ± 0.1* | 56 ± 2* | 89 ± 4* |
| Cd-treated SGECd^t^ | 26 ± 1.0* | 8,0 ± 0.2* | 4,3 ± 0.2* | 72 ± 3* | 62 ± 4* | 28 ± 0.9* | 6,0 ± 0.2^#^ | 114 ± 7^@^ | 38 ± 2* | 18.2 ± 0.6* | 187 ± 16* | 2,9 ± 0.2* | 57 ± 3* | 94 ± 5* |

Different letters show significant differences between treatments within sub columns for untreated and Cd-treated plants, whereas different symbols show significant differences between average values for all inoculation treatments (least significant difference test, *P* < 0.05, n = 5). De data are means ± SE. DW stands for dry weight.

**SUPPLEMENTAL FIGURE S1** Ultrastructural organization of untreated nodules of wild-type SGE inoculated with *R. leguminosarum* bv. *viciae* strain 3841. (**A**) An infected cell from the nitrogen fixation zone. (**B**) An infected cell from the infection zone. ba, bacteroid; cw, cell wall; v, vacuole; a, amyloplast; arrows indicate symbiosome membrane. Scale bar = 500 nm.


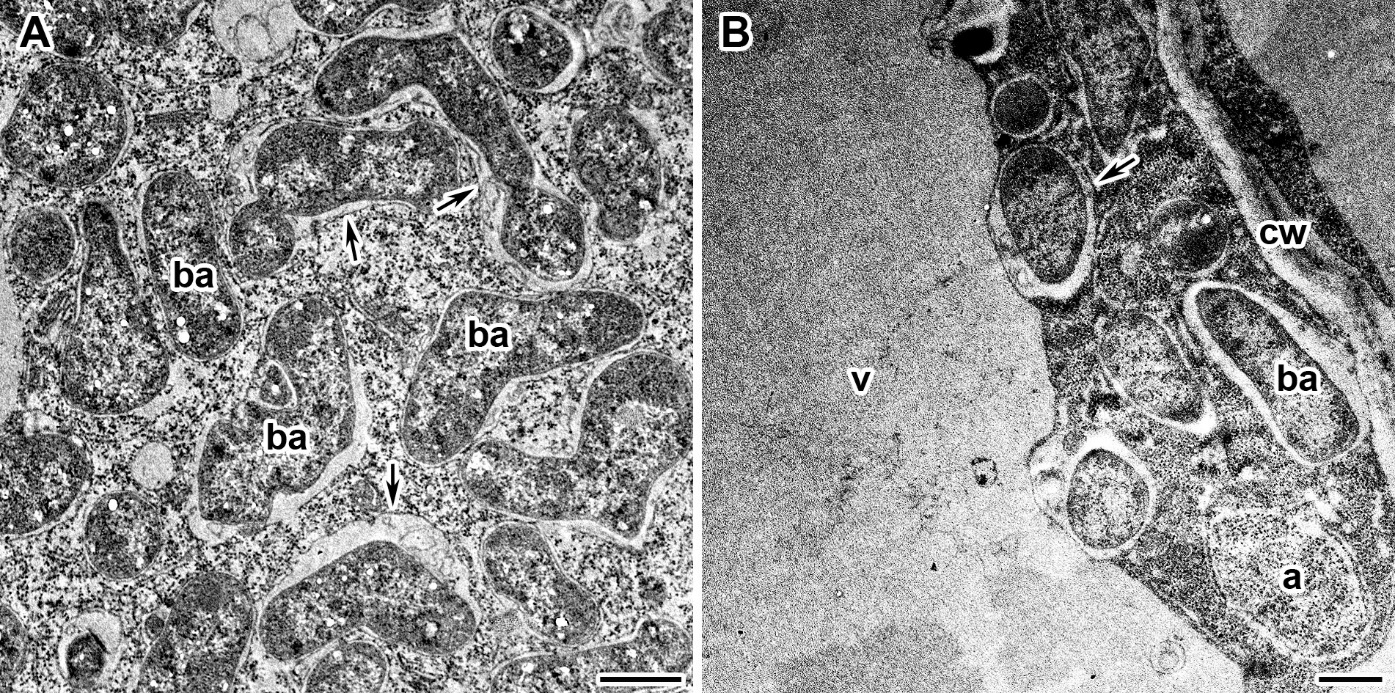


**SUPPLEMENTAL FIGURE S2** Accumulation of the electron dense crystals in vacuole in the cadmium treated nodules of wild-type SGE (**A,C,E**) and mutant SGECd^t^ (**B,D,F**) inoculated with *R. leguminosarum* bv. *viciae* strain 3841 (**A,B**), 3841-MT1 (**C,D**) and 3841-MT2 (**E,F**). ba, bacteroid; b, bacterium; v, vacuole; triangles indicate electron dense crystals in vacuole. Scale bar = 500 nm.


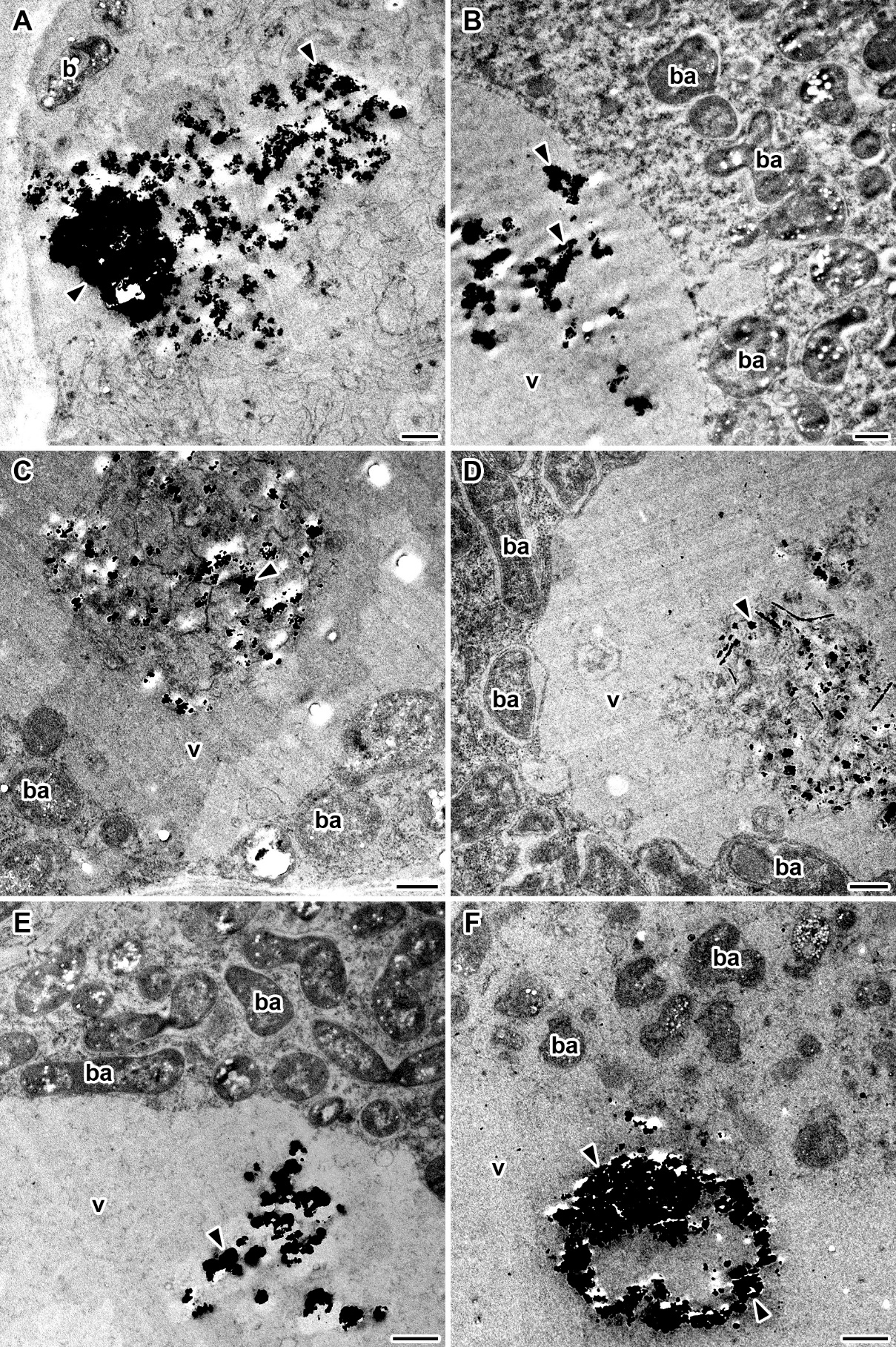

Supplement: Supplementary file 1 [file Data_Sheet_1.docx]
